# Assessing the Impact of Arabinoxylans on Dough Mixing Properties and Noodle-Making Performance through Xylanase Treatment

**DOI:** 10.3390/foods13193158

**Published:** 2024-10-03

**Authors:** Eunbin Ha, Meera Kweon

**Affiliations:** 1 Nutrition Education Major, Department of Education, Pusan National University, Busan 46241, Republic of Korea; ot4321@pusan.ac.kr; 2 Department of Food Science and Nutrition, Pusan National University, Busan 46241, Republic of Korea; 3 Kimchi Research Institute, Pusan National University, Busan 46241, Republic of Korea

**Keywords:** arabinoxylan, wheat flour, xylanase, noodle-making performance, dough property

## Abstract

This study examined the impact of xylanases, focusing on the hydrolysis of water-extractable (WE-AX) and water-unextractable arabinoxylans (WU-AX) and on the quality and noodle-making performance of flours with varying gluten strengths. Flours categorized as strong (S), medium (M), and weak (W) were treated with two xylanases (WE and WU) at concentrations ranging from 0.01% to 0.2%. Parameters such as solvent retention capacity (SRC), SDS sedimentation volume, dough mixing properties, and noodle characteristics were measured. The SRC revealed that flour S had the highest water-holding capacity, gluten strength, and arabinoxylan content. Xylanase treatment reduced water SRC values in flour S and increased the SDS sedimentation volume, with a greater effect from xylanase WU, indicating the potential enhancement of gluten strength. The impact of xylanases was pronounced at higher enzyme concentrations, with differences in dough mixing properties, resistance, and extensibility of fresh noodles, producing softer and stretchable noodles. Cooked noodles made from flours treated with xylanase were softer and had decreased firmness and chewiness, especially those made from flours S and M. This study concludes that WE-AX and WU-AX influence noodle texture; therefore, controlling their degradation with xylanases can produce noodles with varied textures, depending on the gluten strength of the flour.

## 1. Introduction

Wheat is consumed in millions of tons worldwide each year in the form of bread, noodles, cookies, cakes, and pasta [1]. Among these products, noodles are preferred and widely consumed in Asian countries due to their ease of preparation, affordability, and convenience [2]. In Korea, wheat consumption has reached approximately 2 million tons, with an annual per capita consumption of 35.7 kg, making it the second most consumed staple food after rice, which has an annual consumption of 56.4 kg [3,4]. The annual per capita noodle consumption stands at 7.7 kg, equating to consumption once every five days [5]. As consumption increases, factors such as color, nutritional value, and texture have become important considerations in noodle manufacturing [6,7]. Sheeting and cutting are common procedures for developing dough and forming it into strands in Asian noodle production. The sheetability and extensibility of the noodle dough are key factors [8].

Gluten protein, composed of gliadin and glutenin, accounts for 80% of the protein in wheat flour and significantly impacts its functional properties. Gluten determines dough characteristics and the quality of final products such as bread, biscuits, and noodles [9]. The quantity and quality of gluten protein, including the rheological properties of dough and sodium dodecyl sulfate (SDS) sedimentation volume, influence the textural properties of noodles [10,11]. However, unlike bread, noodle dough is prepared using a limited amount of water, which results in minimal gluten development. Therefore, it is necessary to determine optimal water absorption conditions for noodle production [2].

Arabinoxylan (AX) is a non-starch polysaccharide that constitutes 2–3% of wheat flour and is composed of a linear backbone of ß-1,4-linked D-xylopyranosyl units, to which α-L-arabinofuranosyl residues are attached through O-2 and/or O-3 [12]. Arabinoxylan can be classified into water-extractable arabinoxylan (WE-AX) and water-unextractable arabinoxylan (WU-AX), which have different physical and chemical properties, including water absorption [13]. The molecular weight of WU-AX is much larger than that of WE-AX, ranging from 200,000 to 300,000, and it has lower solubility. Additionally, the arabinose substitution level is higher in WU-AX compared to WE-AX. Water absorption is high in AX, and this slows down dough development by disrupting gluten formation and negatively affecting product quality.

Endo-ß-1,4-xylanase (EC 3.2.1.8.), also known as endoxylanase or xylanase, modifies AX and alters its physicochemical properties [13,14]. The enzymes hydrolyze β-1,4-linked bonds in the xylan backbone of arabinoxylan polymers, with different specificities for hydrolyzing WE-AX and WU-AX depending on the type [15]. Both xylanases are used in the baking industry. The former improves cracker quality by reducing water-holding capacity and enhancing dough sheetability through the hydrolysis of WE-AX. The main mechanism of the former involves hydrolyzing WE-AX to produce linear or backbone polymers with a degree of polymerization of less than approximately 100 and, ideally, less than 17 (similar to arabinoxylan–oligosaccharides) [16]. In contrast, the latter improve bread quality by hydrolyzing WU-AX into medium or high molecular weight-solubilized AX, which increases the amount of WE-AX [14]. This increase in WE-AX enhances dough consistency, stiffens the dough, and improves gas retention during baking [17].

Research on the application of xylanase in pasta production has also been conducted. Endoxylanase treatment significantly reduced extrusion pressure during pasta production, maintained the quality of dried pasta, and provided a method for producing high-quality pasta with increased levels of soluble fiber [18]. Xylanase-treated pasta exhibited lower cooking loss and greater firmness compared to the control [19]. However, there is limited research on the impact of xylanase on noodles. Niu et al. [20] used enzymes, including endoxylanase, glucose oxidase, and transglutaminase, in whole-wheat noodles, but the impact of endoxylanase on the quality and sensory properties of the noodles was negligible. Fan et al. [21] reported that reducing the molecular weight of AX through ultrasound treatment, rather than xylanase, positively affected the nutrition and quality of Chinese noodles. Furthermore, no research has reported the role of WE-AX or WU-AX in noodle production through xylanase treatment.

Therefore, this study aims to determine the impact of xylanases that act selectively on WE-AX and WU-AX on flours with different gluten strengths and on noodle-making performance. Two xylanases with different substrate specificities (WE-AX and WU-AX) were examined.

## 2. Materials and Methods

### 2.1. Materials

Three types of commercial wheat flour with different gluten strengths—strong (S), medium (M), and weak (W)—milled with a roller mill were purchased from a local market in Korea. In general, commercial flour S is milled from hard wheat, typically hard red spring wheat from the U.S. and Canada. Flour M is produced from a blend of hard and soft wheat, often hard red winter and soft white from the U.S. or Australian hard and soft white. Flour W is milled from soft wheat, commonly soft white from the U.S. Two types of xylanases, Shearzyme 500 L and Pentopan 500 BG (referred to as xylanase WE and WU, respectively), which act favorably on WE-AX and WU-AX, respectively, were used (Novozymes, Bagsvaerd, Denmark). First-grade reagents were used in all the experiments.

### 2.2. Analyzing Water Solvent Retention Capacity of Flours Treated with Xylanases

Moisture, ash, and protein content of flours were measured according to the AACC Method 44-15.02, 08-01.01, and 46-30.01, respectively [22].

The solvent retention capacity (SRC) of flours was determined according to the AACC Method 56-11.02 [22]; four solvents-distilled water, 5% (*w*/*w*) sodium carbonate solution, 5% (*w*/*w*) lactic acid solution, and 50% (*w*/*w*) sucrose solution-were used to analyze flour quality. To examine the effect of xylanase on the water absorption capacity, the water SRC of flours with different concentrations of xylanase (0.01%, 0.025%, 0.05%, 0.1%, and 0.2% based on flour weight) was measured. The xylanase concentration was chosen based on the recommended doses from a commercial producer of bakery enzymes (xylanase WE, 100–200 mg/kg flour; xylanase WU, 20–180 mg/kg flour) and the previous study by Courtin et al. [14]. This study also tested an expanded range to investigate overdose. The xylanase concentration was chosen based on the recommended doses from a commercial producer of bakery enzymes (xylanase WE, 100–200 mg/kg flour; xylanase WU, 20–180 mg/kg flour) and the previous study by Courtin et al. [14]. This study also tested an expanded range to investigate the effects of overdose.

A 25 mL tube containing flour and water containing xylanases was shaken at 5 min intervals for a total of 20 min to disperse and hydrate the flour and then centrifuged at 1000 × g for 15 min (LaboGene 1248, Gyrozen Inc., Daejeon, Republic of Korea). After centrifugation, the supernatant was discarded, and the pellet weight was measured.

### 2.3. Analyzing Sodium Dodecyl Sulfate Sedimentation Volume of Flours Treated with Xylanases

The gluten strength of the wheat flour was measured by SDS sedimentation volume according to the AACC Method 56-71.01 [22]. Five grams of flour and 50 mL of water containing different concentrations of xylanase were placed in a 100 mL graduated cylinder and shaken vertically and horizontally to hydrate the flour. Subsequently, 50 mL of 3% SDS-lactic acid solution was added and shaken. The graduated cylinder was placed vertically, and the volume (ml) of the flour was measured after 20, 40, and 60 min.

### 2.4. Analyzing the Dough-Mixing Properties of Flours Treated with Xylanases

The dough-mixing properties of flours were determined using a 10 g Mixograph (National Manufacturing Co., Lincoln, NE, USA) according to the AACC Method 54–40.02 [22]. About 10 g of flour and a fixed amount of water for each flour with added xylanases (5.7, 5.9, and 6.8 g for flours S, M, and W, respectively) were placed in a mixograph bowl and mixed for 10 min. Dough mixing patterns were obtained as mixograms, and the peak height and time were analyzed manually based on the maximum point of the mixing curve and the time (in minutes) at the peak.

### 2.5. Preparation of Fresh Noodles Treated with Xylanases

Fresh noodles were prepared from flour based on the method proposed by Wang and Kweon [23]. Flour (100 g), water, or water containing xylanase at different concentrations (32 g) and salt (2 g) were added to a micro-pin mixer (National Manufacturing Co., Lincoln, ME, USA) and mixed for 15 min. The dough was placed in a plastic bag and hydrated for 30 min in an incubator (Phantom M301 Combi, Samjung, Hanam, Republic of Korea) set to 35 °C and 85% relative humidity. The dough was then placed in a noodle maker (SN-88, Samwoo Industrial Co., Daegu, Republic of Korea), sheeted continuously to 3.0, 2.0, and 1.5 mm thickness, and cut to produce fresh noodles (1.5 mm thickness × 3.5 mm width).

### 2.6. Analyzing the Color and Texture Characteristics of Fresh Noodles Treated with Xylanases

The color of fresh noodles was measured using a colorimeter (CR-20, Konica Minolta, Tokyo, Japan) on the dough sheet before cutting it into noodles. The texture of fresh noodles was measured for resistance (R) and extensibility (E) using a texture analyzer (CT3, Brookfield, Middleboro, MA, USA). Ten measurements were performed on individual strands of fresh noodles clamped between the two plates of the Kieffer rig, which were pulled upwards by the hook until a fracture occurred. The measurement conditions were as follows: probe: Kieffer rig (TA-KF); test mode: tension; target value: 20 mm; pre-test speed: 2 mm/s; and test speed: 3.3 mm/s.

### 2.7. Analyzing the Cooking Properties and Texture Characteristics of Cooked Noodles Treated with Xylanases

To evaluate the quality of cooked noodles, 15 g of fresh noodles were placed in 500 mL of boiling water and cooked for 15 min. The turbidity of the cooking water was determined by measuring the absorbance at 625 nm using a spectrophotometer (X-ma 61,100 PC; Human Corporation, Seoul, Republic of Korea). The weight gain of cooked noodles was calculated by measuring weight before and after boiling.

The texture of the cooked noodles was analyzed using a texture analyzer (CT3, Brookfield). After rinsing the cooked noodles with tap water and removing excess water, five strands of noodles were aligned on a texture analyzer plate. Firmness, adhesiveness, resilience, cohesiveness, springiness, and chewiness of cooked noodles were measured. The measurement conditions were as follows: probe, Asian noodle ring (TA 7); test mode, TPA; deformation, 70%; pretest speed, 2 mm/s; test speed, 1 mm/s; and posttest speed, 1 mm/s.

### 2.8. Statistical Analysis

All data were obtained from at least four measurements from duplicate experiments. Variations in flour and noodle quality were analyzed using Tukey’s HSD test with SPSS Statistics (version 27.0, IBM, Armonk, NY, USA). Statistically significant differences were verified at the *p* < 0.05 level.

## 3. Results and Discussion

### 3.1. Water SRC of Flour Treated with Xylanases

Table 1 shows the moisture, ash, and protein content, along with the SRC values of flours with different gluten strengths. The moisture content of the tested flours ranged from 13.1% to 14.1%, which was close to the desirable range of 13–14% for producing wheat-based products, including bread, pasta, noodles, cookies, and crackers [24]. The ash content of these flours was between 0.52% and 0.55%, with no significant differences observed among the three flours (*p* > 0.05). Commercial flour milling aims to extract the starchy endosperm from the grain and produce flour with low levels of bran and germ, thereby lowering the ash content [25]. The ash content of commercial white winter wheat flour is approximately 0.5% [26], comparable to that of flours used in this study.

The protein content of the flour samples ranged from 8.9% to 13.4% (based on 14% moisture content), in the order W < M < S (Table 1). Flour S, milled primarily from hard wheat, typically has a high protein content (10.0–14.0%). In contrast, flour W, milled mainly from soft wheat, has a low protein content (8.5–10.5%) [24]. Flour M is made from a blend of hard and soft wheat. This variation reflects the differences in protein content, as well as the blending ratio of hard and soft wheat, which influences the gluten strength of the flours.

Water hydrates and swells the functional components of flour, such as gluten, damaged starch, and arabinoxylans. Each functional component exaggeratedly swells in a specific solvent. The water SRC value reflects the flour water-holding capacity, which is a crucial characteristic related to processing and final product quality [24]. The water SRC values for flours S, M, and W are 71.6%, 64.1%, and 52.8%, respectively. Flour S had the highest water SRC, whereas flour W had the lowest. In flour, arabinoxylans, damaged starch, and gluten protein contribute to water-holding capacity. The water SRC results suggested higher levels of these components in flour S than in flour W. The sodium carbonate SRC values for flours S, M, and W were 95.8%, 83.7%, and 68.9%, respectively, indicating a higher contribution of damaged starch in flour S than in flours M and W. The lactic acid SRC values for flours S, M, and W were 156.6%, 124.2%, and 98.7%, respectively, confirming that flour S had the highest gluten strength, and flour W had the lowest gluten strength (*p* < 0.05). Courtin and Delcour [27] reported that a weak AX gel network could reinforce the gluten network through the high molecular weight of WE-AX. Flour S can be expected to develop a stronger gluten network than flour W with xylanase treatment. The sucrose SRC values for flours S, M, and W were 126.0%, 112.0%, and 99.3%, respectively, confirming the highest arabinoxylan contribution in flour S and the lowest in flour W (*p* < 0.05). Flour S may be more affected by xylanases than flour W. The SRC values for all four solvents followed the same order: W < M < S.

Based on the SRC values of the lactic acid, sodium carbonate, and sucrose solutions, the calculated gluten performance index (GPI) values of flours S, M, and W were 0,71, 0.63, and 0.59, respectively, which followed the same order as the SRC values (W < M < S, *p* < 0.05). The GPI represents the overall performance of glutenin [24] and indicates that flour S has a higher glutenin performance than flour W.

**Figure 1:**
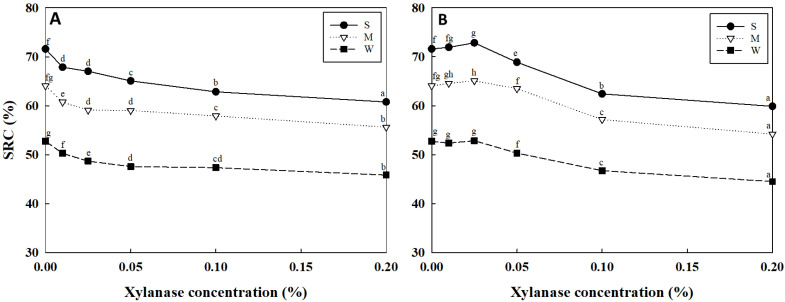
Changes in the water SRC of flours treated with xylanases at different concentrations. (**A**) Xylanase WE (**B**) Xylanase WU. The different letters above the symbols (means from *n* = 4) within the same flour, treated with two xylanases in both graphs, indicate significant differences (*p* < 0.05) according to Tukey’s HSD test.

Figure 1 shows the water SRC values of the flours treated with the two xylanases at different concentrations.

When treated with xylanase WE, the water SRC values for flour S ranged from 71.6% to 60.8%, for flour M from 64.1% to 55.6%, and for flour W from 50.3% to 45.8%, with the largest decrease in flour S and least in flour W. These results may be due to differences in the swellable arabinoxylans in each flour, as demonstrated by their sucrose SRC values. As the xylanase concentration increased, the water SRC values decreased. This decrease was due to increased hydrolysis of AXs, which reduced their molecular weight and consequently affected the water absorption capacity (*p* < 0.05). Generally, WE-AX is known for its high water-holding capacity, ability to form a network in dough that increases viscosity, and resistance to spread and expansion [17]. Therefore, the addition of xylanase WE, which degrades WE-AX, significantly decreased water SRC, resulting in reduced water absorption [23,28]. Moon and Kweon [29] also reported that a 0.025% increase in xylanase concentration led to a decrease in the water SRC value on the addition of xylanase and amylase to dry noodle production, confirming a trend consistent with the results of this study.

In comparison, when treated with xylanase WU, the water SRC values for flour S ranged from 72.8% to 59.9%, for flour M from 65.1% to 54.2%, and for flour W from 52.8% to 44.5%. These changes were similar to those observed for xylanase WE, although the decreases were marginally greater. The effect of xylanase WU on WU-AX degradation was more pronounced, as 70–75% of wheat arabinoxylan consists of WU-AX, while 25–30% consists of WE-AX [27]. As the xylanase concentration increased up to 0.025%, the decrease in the water SRC value was not significant; however, further increases in the xylanase concentration caused a rapid decrease (*p* < 0.05). Overall, the two xylanases functioned differently, leading to distinct patterns in the change in the water SRC values depending on the xylanase concentration.

### 3.2. SDS Sedimentation Volume of Flours Treated with Xylanases

**Figure 2:**
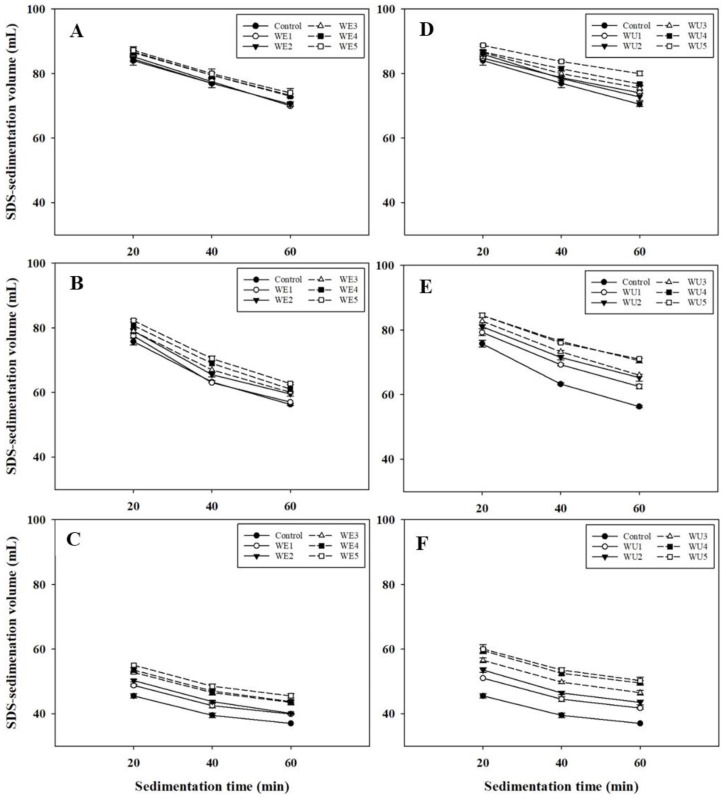
Changes in SDS-sedimentation volume of flours treated with S and P xylanases during the sedimentation period. (**A**,**C**,**E**) Flour S, M, and W with xylanase WE; (**B**,**D**,**F**) Flour S, M, and W with xylanase WU.

Figure 2 shows the SDS sedimentation volume results for flours treated with different concentrations of xylanases. As the sedimentation time increased, the SDS sedimentation volumes decreased, and this decreasing pattern depended on the flour type; flour S showed a continuous decrease, whereas flour W exhibited a slower decrease.

When flour S was treated with xylanase WE, the SDS sedimentation volumes at 20, 40, and 60 min were 84.0~87.3 mL, 77.0~80.0 mL, and 70.5~74.0 mL, respectively. With increasing xylanase concentrations, there was no significant difference at 20 and 40 min, but a slight increase was observed at 60 min. In comparison, the SDS sedimentation volumes of flour S treated with xylanase WU at 20, 40, and 60 min were 84.0~88.8 mL, 77.0~83.8 mL, and 70.5~80.0 mL, respectively, showing a slight increase at 20 min and significant increases at 40 and 60 min with increasing xylanase concentration (*p* < 0.05).

For flour M treated with xylanase WE, the SDS sedimentation volumes at 20, 40, and 60 min were 75.8~82.3 mL, 63.3~70.5 mL, and 56.3~62.8 mL, respectively, showing significant increases as the xylanase concentration increased. In comparison, the SDS sedimentation volumes of flour M treated with xylanase WU at 20, 40, and 60 min were 75.8~84.5 mL, 63.3~76.0 mL, and 56.3~71.0 mL, respectively. These also showed significant increases with increasing xylanase concentrations, with xylanase WU causing a much higher increase than xylanase WE.

For flour W, which had the lowest gluten strength in this study, treatment with either xylanase WE or WU resulted in significant increases in the SDS sedimentation volume with increasing xylanase concentration, although the impact of xylanase WU was more pronounced. The SDS sedimentation volumes of flour W treated with xylanase WE at 20, 40, and 60 min were 45.5~55.0 mL, 39.5~48.5 mL, and 37.0~45.5 mL, respectively. In comparison, the volumes with xylanase WU at 20, 40, and 60 min were 45.5~60.0 mL, 39.5~53.5 mL, and 37.0~50.3 mL, respectively.

Overall, flour S had the highest SDS sedimentation volume, whereas flour W showed the lowest, indicating distinct gluten strength in the following order: W < M < S. As the concentration of xylanase increased, the SDS sedimentation volumes for all the sampled flours increased. Xylanase WU had a significantly greater impact on SDS sedimentation volume than xylanase WE. This may be explained by the fact that WU-AX is more firmly bound to the cell wall surface and interacts, both covalently and non-covalently, with other AX molecules or proteins, lignin, or cellulose, compared to WE-AX [27]. Xylanase treatment facilitates the glutenin swelling, with the hydrolysis of WU-AX having a greater effect than WE-AX. Additionally, previous studies reported that xylanase improved gluten aggregation and production by hydrolyzing arabinoxylans [30,31]. Xylanase treatment decreases water absorption attributed to arabinoxylans in flour, thereby providing sufficient water for the development of gluten proteins that can positively affect gluten formation.

The slope obtained from the changes in SDS sedimentation volumes from 20 to 60 min, which indicates gluten instability, increased in the following order: S (0.16) < W (0.19) < M (0.26). With xylanase treatment, the slope values decreased, with xylanase WU treatment showing a significant decrease than xylanase WE, indicating an increase in gluten stability.

Table 2 shows the relative percentage increase in SDS sedimentation volumes for flours S, M, and W with varying gluten strengths when treated with either xylanase WE or WU, compared to those without xylanases. The addition of xylanases resulted in the highest increase in SDS sedimentation volume in flour W and the lowest in flour S, with a much greater increase observed after the addition of xylanase WU. These results suggest that xylanase has a more pronounced effect on the swellability of glutenin in flours with lower gluten strength in SDS-lactic acid solution, likely due to a weaker interaction between gluten proteins and arabinoxylans compared to flours with higher gluten strength [32]. Additionally, xylanase significantly impacts the hydrolysis of WU-AX.

### 3.3. Dough Mixing Property of Flours Treated with Xylanases

**Figure 3:**
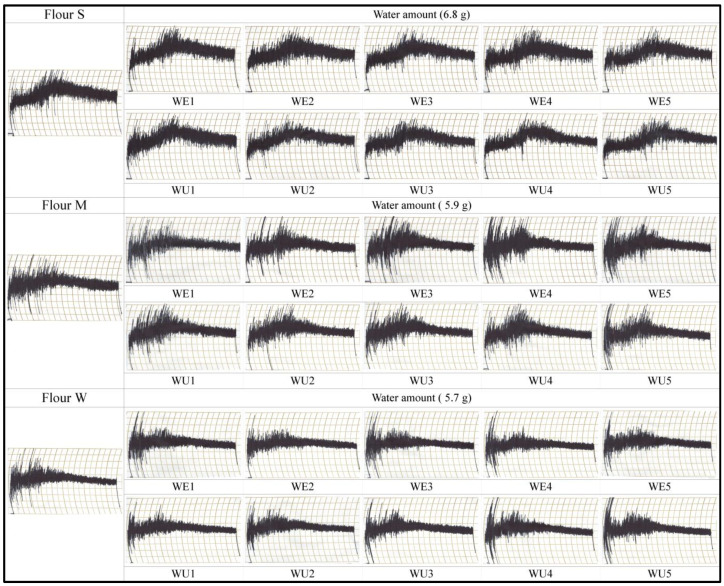
Mixograms of flour treated with xylanases WE and WU.

Figure 3 shows the mixograms of flours treated with xylanases at various concentrations. For flour S treated with xylanase, the bandwidth narrowed more significantly with xylanase WU than with xylanase WE, as the xylanase concentration increased. The xylanase-treated dough became less dry owing to the decreased water absorption capacity, as indicated by the water SRC results, leading to reduced competition for water [27,33]. Xylanase WU had a more pronounced impact on dough mixing properties than xylanase WE. Additionally, as the xylanase concentration increased, the peak time for dough development was delayed owing to the increased availability of water [34]. Mohammadi et al. [35] also reported a positive effect of xylanase addition on gluten formation in flour dough.

The mixing bandwidth of flour M was narrower than that of flour S, indicating weaker gluten strength. The slope of the band after the mixing peak showed a gradual decrease. For flour M, as the xylanase concentration increased, the bandwidth after the mixing peak became narrower, with xylanases WE and WU exhibiting similar effects.

The mixograms of flour W displayed a noticeably narrower band than those of flours S and M, reflecting the weakest gluten strength among the sampled flours. However, with the xylanase treatment, the dough-mixing patterns of flour W showed less pronounced changes than those of flours S and M.

### 3.4. Color and Texture of Fresh Noodles

Table 3 shows the color of fresh noodles made from flours treated with different concentrations of xylanases. Noodles prepared from flour S appeared darker (low L* values) and more reddish (high a* values), whereas those prepared from flour W were brighter (high L* values) and more yellowish (high b* values). For noodles made from all flour, the L* values for samples treated with xylanase WE and WU were lower than that of the control, indicating that xylanase treatment increased darkness. However, there was no significant difference or only a slight decrease in L* values as the concentration of xylanase WE or WU increased. This trend is consistent with the observed decrease in the L* value of whole wheat noodles when xylanase was added [20]. The a* and b* values for noodles made from all flour with xylanase treatment were similar to or higher than that of the control, with no significant changes observed as the xylanase concentration increased. These results suggest that the reduced water absorption capacity due to xylanase addition, similar to the impact of increased water addition, could influence noodle color. Hatcher et al. [36] reported that increased water addition decreases the L* value and increases the b* value of noodles. Polyphenol oxidase (PPO) in flour darkens fresh noodles [37]. Xylanase treatment may enhance PPO activity by interacting with phenolic substrates such as ferulic acid in arabinoxylans [20].

Table 4 lists the resistance, extensibility, and R/E ratios of fresh noodles made from flours treated with different xylanase concentrations. Resistance indicates the force required to stretch the dough, whereas extensibility reflects the extent to which the dough can stretch before breaking [38]. The resistance and extensibility of fresh noodles increased in the following order: NW < NM < NS.

For noodles made from flour S, the resistance values of NS-WE1 through NS-WE5 (1.94~2.08 N) and those of NS-WU1 through NS-WU5 (1.64~1.85 N) were not significantly different from NS-C (2.01 N). However, the extensibility values for NS-WE1 through NS-WE5 (10.0~10.2 mm) and NS-WU1 through NS-WU5 (9.4~10.6 mm) were significantly higher compared to NS-C (9.6 mm) (*p* < 0.05). The R/E ratios showed no significant variation with increasing xylanase WE concentration, but for NS-WU1 through NS-WU5, the R/E ratios were 0.16 to 0.20, decreasing as the concentration of xylanase WU increased. These results suggest that xylanase WU had a more pronounced effect on resistance than on extensibility. The addition of xylanase WE to flour S increased extensibility, resulting in noodles that could stretch well. Conversely, xylanase WU reduced resistance and increased extensibility, producing softer and easier-to-stretch noodles. The quality of noodles is influenced by the amount of water available during dough preparation. Insufficient water hardens the dough, whereas excessive water results in a loose and overly stretched dough [39]. These results indicate that the hydrolysis of WE-AX and WU-AX during noodle making can affect the texture of fresh noodles.

For noodles made from flour M, the resistance values for NM-WE1 through NM-WE5 (1.10~1.37 N) and NM-WU1 through NM-WU5 (1.04~1.18 N) were lower than that of NM-C (1.21 N). The extensibility values of NM-WE1 through NM-WE5 (6.2~7.7 mm) and those of NM-WU1 through NM-WU5 (6.2~7.5 mm) were similar to or higher than that of NM-C (6.6 mm). The addition of xylanase resulted in softer and more easily stretched noodles. The R/E ratios of NM showed a similar trend to those observed for NS.

For noodles made from flour W, the resistance values of NW-WE1 through NW-WE5 (0.53~0.67 N) and those of NW-WU1 through NW-WU5 (0.43~0.67 N) were significantly lower than NW-C (0.75 N), indicating a similar impact of xylanases on resistance. However, the effect on the extensibility varied depending on the type of xylanase used. The extensibility of NW-WE1 through NW-WE5 ranged from 2.7 to 3.1 mm, which was significantly lower than that of NW-C (4.0 mm) (*p* < 0.05). In contrast, the extensibility of NW-WU1 through NW-WU5 ranged from 4.1 to 5.2 mm, which was significantly higher than that of NW-C (*p* < 0.05). The addition of xylanase WU resulted in softer and more easily stretched noodles, indicating that the hydrolysis of WU-AX is pronounced in noodles made from flours with weak gluten strength. The R/E ratio ranged from 0.11 to 0.13 in NW-WU1 through NW-WU5, significantly lower than that of NW-C (*p* < 0.05).

Overall, the resistance and extensibility of fresh noodles were highest in NS, which has strong gluten strength, and lowest in NW, which has weak gluten strength. This suggests that stronger gluten results in noodles that are firmer and better stretched. The addition of more xylanase WE and WU decreased the resistance and increased extensibility, indicating that the hydrolysis of both WE-AX and WU-AX led to softer and more extensible noodles. This effect was particularly pronounced during the hydrolysis of WU-AX. Specifically, the solubilization of WU-AX by xylanase reduced the water-holding capacity of the flour, resulting in the redistribution of previously bound water in gluten and increased dough extensibility [27]. During noodle production, the sheeting and cutting processes require dough that is well-balanced with both good extensibility and resistance to form smooth noodle sheets and prevent breakage. This balance requires a certain level of gluten strength [19,40,41]. Therefore, xylanase addition can alter dough resistance and extensibility, indicating its potential use in the production of noodles with various textures.

### 3.5. Quality Characteristics of Cooked Noodles

**Figure 4:**
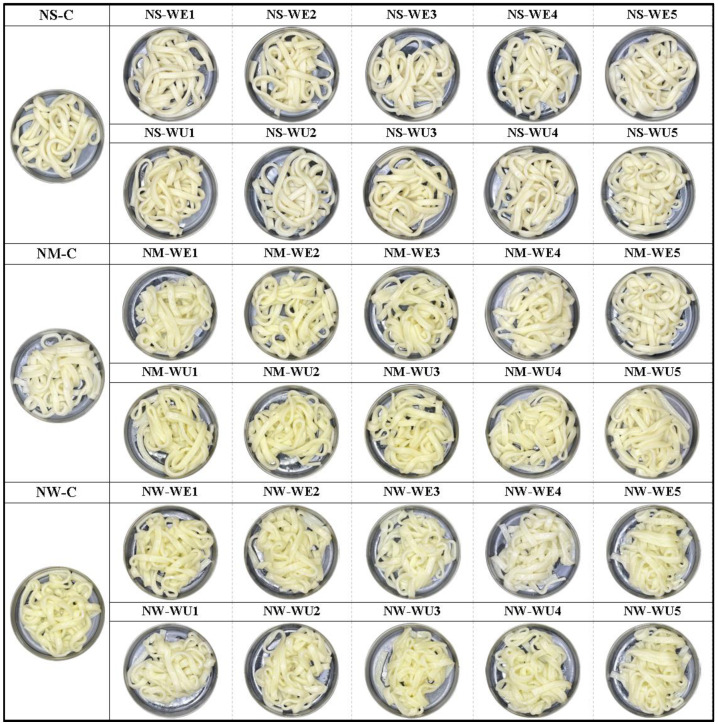
Appearance of cooked noodles made with flours treated with xylanases WE and WU.

Figure 4 shows the appearance of cooked noodles. It can be observed that NS is relatively thick, opaque, and bouncy, whereas NW is thin, translucent, and mushy, indicating that gluten strength influences noodle characteristics. When xylanase was added, the color of fresh noodles darkened, as shown in the color data in Table 3. However, no significant difference was observed in the color of the cooked noodles.

Table 5 lists weight gain and turbidity observed in the cooking water for noodles made from different flours. Weight gain increased in the order of NS < NM < MW, whereas turbidity of the cooking water increased in the order NM < NW < NS. After xylanase treatment, NS and NM did not show a significant increase in weight gain, whereas NW did. Turbidity of the cooking water increased for all noodles treated with xylanase, with both xylanases showing similar effects. This result suggests that solid materials, such as starch and proteins, leached into the water during cooking because xylanases may loosen the AX-starch or AX-protein structure.

Table 6 shows that firmness and chewiness of cooked noodles increased in the order of NW < NM < NS, while adhesiveness increased in the order of NW ≤ NM < NS. For all noodles, firmness, adhesiveness, and chewiness decreased as the concentration of xylanase increased. For noodles made from flour S, the firmness of NS-WE1~NS-WE5 (15.9~18.4 N) and NS-WU1~NS-WU5 (14.6~18.3 N) was lower than that of NS-C (18.7 N). Increasing the concentrations of xylanases WE and WU decreased firmness (*p* < 0.05). This result can be explained by the hydrolysis of WE-AX and WU-AX, which reduces water absorption during dough development, leading to softer cooked noodles. Park and Baik [2] found that water absorption impacted the firmness of both fresh and cooked noodles. Noodles made from flours M and W exhibited similar trends. The firmness of NM-WE1~NM-WE5 (11.4~14.9 N) and NM-WU1~NM-WU5 (9.5~13.0 N) was significantly lower than that of NM-C (15.3 N). As the concentrations of xylanases WE and WU increased, firmness decreased significantly (*p* < 0.05). The firmness of NW-WE1~NW-WE5 (10.6~11.6 N) and NM-WU1~NM-WU5 (10.2~11.5 N) was also much lower compared to that of NM-C (12.2 N), with firmness decreasing as the concentration of xylanases WE and WU increased (*p* < 0.05).

The adhesiveness of NS-WE1~NS-WE5 (0.25~0.28) and NS-WU1~NS-WU5 (0.21~0.29) was similar to or lower than that of NS-C (0.29). Adhesiveness, which represents the force required to separate adhered noodles from the test probe and indicates the stickiness/smoothness characteristic of food in sensory evaluations, decreased with the addition of xylanase WU [42,43]. This shows that the addition of xylanase WU results in less sticky but smoother noodles. The adhesiveness of NM-WU5 was significantly lower at 0.16 compared to NM-C at 0.21. The adhesiveness of NW-WE5 was 0.15, lower than that of NW-C (0.20) (*p* < 0.05).

The chewiness of NS-WE1~NS-WE5 (8.5~9.9 mJ) and NS-WU1~NS-WU5 (8.1~9.6 mJ) was lower than that of NC (10.2 mJ) and decreased significantly with higher concentrations of xylanase WE and WU (*p* < 0.05). Chewiness refers to the mouthfeel sensation experienced during the chewing of food, resulting from its sustained and elastic resistance [44], following a trend similar to that of firmness. The chewiness of NM-WE1~NM-WE5 (6.4~7.7 mJ) and NM-WU1~NM-WU5 (5.3~7.0) was significantly lower than that of NM-C (8.3) (*p* < 0.05), verifying the hydrolysis effect of xylanase WE and WU on WE-AX and WU-AX. The chewiness of NW-WE1~NW-WE5 (5.5~6.5 mJ) and NM-WU1~NM-WU5 (5.2~6.1 mJ) was lower than that of NW-C (6.9 mJ).

Table 7 lists the resilience, cohesiveness, and springiness of cooked noodles. The resilience of cooked noodles increased in the order of NW < NM ≤ NS, whereas cohesiveness and springiness did not show significant differences among flour types. Additionally, the resilience, cohesiveness, and springiness of cooked noodles did not vary with xylanase concentration. Hou et al. [45] reported that firmness, springiness, and chewiness of noodles showed a negative correlation with the extensibility of the dough but a positive correlation with the resistance of dough, reflecting a trend similar to the findings of this study.

Numerous reports have highlighted the beneficial impacts of xylanases on bread quality, including increased loaf volume, improved bread-making scores, finer and softer crumb structure, and extended shelf life [14,27,46,47,48]. This study suggests the potential application of xylanase for producing noodles with diverse textures.

## 4. Conclusions

This study investigated the impact of xylanases, specifically the hydrolysis of WE-AX and WU-AX, on the quality characteristics of flours with different gluten strengths and noodle-making performance. Commercial flours with varying gluten strengths (S, M, and W) were treated with two types of xylanases (WE and WU) at concentrations ranging from 0.01% to 0.2%.

The solvent retention capacity (SRC) analysis confirmed that flour S had the highest values among all solvents, indicating a higher water-holding capacity, greater damaged starch content, stronger gluten strength, higher arabinoxylan (AX) content, and a higher gluten performance index. Both xylanases reduced the water SRC values as their concentrations increased, with flour S exhibiting the most significant decrease. The increase in SDS sedimentation volume was highest in flour W and lowest in flour S, with xylanase WU displaying a more pronounced effect on gluten development, particularly in flours with weaker gluten strength.

Xylanase treatment, especially with xylanase WU, affected dough mixing properties and fresh noodle characteristics, including resistance, extensibility, and color; high concentrations of xylanases WE and WU decreased the resistance of fresh noodles while increasing extensibility, resulting in softer and more stretchable noodles. Characteristics such as firmness, adhesiveness, and chewiness of cooked noodles reduced significantly following xylanase treatment, confirming the impact of AX hydrolysis on noodle texture, with a greater effect resulting from WU-AX degradation. The impact of xylanase treatment was higher for noodles made from flour S than for noodles made from flours M and S.

Overall, this study shows that xylanase treatment alters the texture of fresh and cooked noodles by reducing water absorption and influencing gluten development through the hydrolysis of AXs in flour. The extent of these changes varies depending on the gluten strength of the flour and the type of xylanase, whether it degrades WE-AX or WU-AX, resulting in noodles with a range of textures. Additionally, adjusting the water amount in flour with xylanase treatment for noodle production, particularly for making dry noodles, would be worth investigating in the future.

## Figures and Tables

**Table 1:** Moisture, ash, and protein content, solvent retention capacity, and gluten performance index of flours.

Flour	Moisture Content (%)	Ash Content (%)	Protein Content (%)	SRC (%)				GPI ^(2)^
				Water	Lactic Acid	Sodium Carbonate	Sucrose	
S	14.1 ± 0.1 ^c(1)^	0.54 ± 0.02 ^a^	13.4 ± 0.0 ^c^	71.6 ± 0.5 ^c^	156.6 ± 0.2 ^c^	95.8 ± 0.3 ^c^	126.0 ± 0.4 ^c^	0.71 ± 0.00 ^c^
M	13.7 ± 0.1 ^b^	0.55 ± 0.01 ^a^	10.7 ± 0.0 ^b^	64.1 ± 0.2 ^b^	124.2 ± 0.2 ^b^	83.7 ± 0.4 ^b^	112.0 ± 0.2 ^b^	0.63 ± 0.00 ^b^
W	13.1 ± 0.1 ^a^	0.52 ± 0.03 ^a^	8.9 ± 0.1 ^a^	52.8 ± 0.2 ^a^	98.7 ± 0.7 ^a^	68.9 ± 0.5 ^a^	99.3 ± 0.5 ^a^	0.59 ± 0.00 ^a^

^(1).^ Means (*n* = 6) with different letters within the same column are significantly different (*p* < 0.05), according to Tukey’s HSD test. ^(2).^ GPI: gluten performance index = LA SRC/(SC SRC + SU SRC).

**Table 2:** Relative percentage increase in SDS sedimentation volume of each flour treated with the same xylanase.

Flour	Xylanase	Increase in SDS Sedimentation Volume (%)		
		20 min	40 min	60 min
S	WE	2.5 ± 1.9 ^a(1)^	2.2 ± 2.2 ^a^	2.3 ± 2.7 ^a^
	WU	3.1 ± 2.3 ^ab^	4.6 ± 3.2 ^a^	7.5 ± 3.9 ^ab^
M	WE	5.2 ± 2.4 ^ab^	5.9 ± 4.4 ^a^	6.8 ± 3.6 ^a^
	WU	8.8 ± 3.0 ^bc^	15.9 ± 4.6 ^b^	19.2 ± 6.2 ^cd^
W	WE	14.4 ± 5.3 ^c^	15.6 ± 6.2 ^b^	15.0 ± 6.3 ^bc^
	WU	23.3 ± 8.1 ^d^	24.9 ± 9.4 ^c^	25.1 ± 9.5 ^d^

^(1)^ Means (*n* = 4) with different lowercase letters within the same column vary significantly (*p* < 0.05), according to Tukey’s HSD test.

**Table 3:** Color of fresh noodles made from flours treated with xylanases.

Sample	L*			a*			b*		
	NS	NM	NW	NS	NM	NW	NS	NM	NW
C	83.4 ± 1.3 ^dA(1)^	84.1 ± 0.1 ^eAB^	84.9 ± 0.5 ^efB^	4.4 ± 0.4 ^aB^	3.8 ± 0.1 ^abA^	4.2 ± 0.2 ^cdeAB^	17.7 ± 1.4 ^aA^	19.2 ± 0.6 ^abB^	22.5 ± 0.9 ^abcC^
WE1	81.8 ± 0.6 ^bcA^	84.6 ± 0.8 ^eB^	84.7 ± 0.3 ^defB^	4.8 ± 0.2 ^abcC^	3.7 ± 0.2 ^aA^	4.4 ± 0.1 ^defB^	19.2 ± 0.6 ^abA^	19.6 ± 0.9 ^abA^	22.8 ± 0.7 ^abcdeB^
WE2	81.0 ± 0.5 ^abcA^	80.9 ± 0.8 ^abcA^	84.0 ± 0.6 ^abcdB^	4.8 ± 0.2 ^abcC^	4.0 ± 0.1 ^abcdA^	4.5 ± 0.1 ^fB^	19.3 ± 0.4 ^abA^	19.3 ± 0.8 ^abA^	23.9 ± 0.5 ^deB^
WE3	82.2 ± 0.3 ^cdA^	81.7 ± 0.7 ^bcA^	84.2 ± 0.6 ^bcdeB^	4.7 ± 0.1 ^abcB^	4.3 ± 0.2 ^dA^	4.5 ± 0.1 ^efAB^	18.7 ± 0.4 ^abA^	19.6 ± 1.1 ^abA^	24.0 ± 0.4 ^eB^
WE4	82.4 ± 0.6 ^cdA^	81.4 ± 1.0 ^bcA^	84.7 ± 0.2 ^defB^	4.7 ± 0.2 ^abcC^	3.8 ± 0.3 ^abA^	4.2 ± 0.1 ^cdeB^	19.5 ± 1.0 ^abA^	18.4 ± 1.9 ^abA^	23.6 ± 0.4 ^cdeB^
WE5	81.6 ± 0.5 ^bcB^	79.8 ± 0.8 ^aA^	85.1 ± 0.2 ^fC^	4.9 ± 0.3 ^abcB^	4.0 ± 0.1 ^bcdA^	4.1 ± 0.1 ^cdA^	19.8 ± 1.2 ^bA^	20.1 ± 0.4 ^bA^	22.7 ± 0.5 ^abcdB^
WU1	81.7 ± 1.3 ^bcA^	83.8 ± 0.5 ^deB^	84.4 ± 0.4 ^cdefB^	4.6 ± 0.4 ^abcB^	3.9 ± 0.2 ^abcA^	3.8 ± 0.1 ^abcA^	18.6 ± 1.4 ^abA^	18.8 ± 1.1 ^abA^	22.2 ± 1.0 ^abB^
WU2	81.1 ± 0.6 ^abcA^	84.1 ± 0.3 ^eB^	84.0 ± 0.6 ^abcdB^	4.9 ± 0.2 ^abcB^	3.8 ± 0.1 ^abA^	3.7 ± 0.3 ^abA^	19.7 ± 0.8 ^abA^	19.1 ± 0.9 ^abA^	21.8 ± 0.9 ^aB^
WU3	80.4 ± 0.7 ^abA^	82.3 ± 0.4 ^cdB^	83.8 ± 0.3 ^abcC^	4.5 ± 0.6 ^abB^	4.2 ± 0.2 ^cdAB^	3.7 ± 0.3 ^aA^	19.0 ± 1.2 ^abA^	19.6 ± 0.5 ^abA^	22.4 ± 0.8 ^abcB^
WU4	79.8 ± 1.0 ^aA^	80.8 ± 0.6 ^abA^	83.4 ± 0.3 ^abB^	5.0 ± 0.3 ^bcB^	4.1 ± 0.2 ^bcdA^	4.1 ± 0.1 ^bcdA^	19.3 ± 1.3 ^abA^	19.3 ± 0.7 ^abA^	23.2 ± 0.5 ^bcdeB^
WU5	79.7 ± 0.8 ^aA^	80.7 ± 1.3 ^abA^	83.2 ± 0.4 ^aB^	5.1 ± 0.2 ^cB^	3.8 ± 0.1 ^abA^	4.0 ± 0.1 ^abcA^	19.4 ± 1.1 ^abB^	17.9 ± 0.7 ^aA^	22.8 ± 0.5 ^abcdeC^

^(1)^ Means (*n* = 6) with different lowercase letters within the same column and those with different uppercase letters within the same row for the same parameter are significantly different (*p* < 0.05), according to Tukey’s HSD test.

**Table 4:** Texture of fresh noodles made from flours treated with xylanases.

Sample	Resistance (N)			Extensibility (mm)			R/E		
	NS	NM	NW	NS	NM	NW	NS	NM	NW
C	2.01 ± 0.08 ^deC(1)^	1.21 ± 0.05 ^cB^	0.75 ± 0.02 ^gA^	9.6 ± 0.5 ^abC^	6.6 ± 0.3 ^abB^	4.0 ± 0.2 ^cA^	0.21 ± 0.01 ^eB^	0.18 ± 0.01 ^deA^	0.19 ± 0.01 ^dA^
WE1	2.02 ± 0.03 ^deC^	1.18 ± 0.05 ^cB^	0.67 ± 0.01 ^fA^	10.2 ± 0.3 ^bcC^	6.2 ± 0.5 ^aB^	2.9 ± 0.1 ^abA^	0.20 ± 0.01 ^deA^	0.19 ± 0.02 ^eA^	0.23 ± 0.01 ^fB^
WE2	1.94 ± 0.10 ^cdC^	1.37 ± 0.04 ^dB^	0.54 ± 0.02 ^bcA^	10.2 ± 0.3 ^bcC^	7.6 ± 0.4 ^cdB^	2.8 ± 0.1 ^abA^	0.19 ± 0.01 ^cdAB^	0.18 ± 0.01 ^cdeA^	0.19 ± 0.01 ^dB^
WE3	2.00 ± 0.07 ^deC^	1.17 ± 0.03 ^cB^	0.53 ± 0.01 ^bA^	10.0 ± 0.5 ^abcC^	7.1 ± 0.3 ^bcdB^	3.1 ± 0.2 ^bA^	0.20 ± 0.02 ^deB^	0.16 ± 0.01 ^abcA^	0.17 ± 0.01 ^cA^
WE4	2.08 ± 0.07 ^eC^	1.16 ± 0.05 ^cB^	0.56 ± 0.02 ^cdA^	10.1 ± 0.5 ^bcC^	7.7 ± 0.5 ^dB^	3.1 ± 0.1 ^bA^	0.21 ± 0.01 ^deC^	0.15 ± 0.01 ^aA^	0.18 ± 0.01 ^cdB^
WE5	1.95 ± 0.08 ^dC^	1.10 ± 0.04 ^bB^	0.57 ± 0.02 ^cdA^	10.0 ± 0.2 ^abcC^	7.1 ± 0.3 ^bcB^	2.7 ± 0.2 ^aA^	0.20 ± 0.01 ^deB^	0.16 ± 0.00 ^abA^	0.21 ± 0.02 ^eC^
WU1	1.85 ± 0.07 ^bcC^	1.16 ± 0.04 ^cB^	0.67 ± 0.01 ^fA^	9.4 ± 0.5 ^aC^	6.2 ± 0.4 ^aB^	5.2 ± 0.3 ^eA^	0.20 ± 0.02 ^deB^	0.19 ± 0.02 ^eB^	0.13 ± 0.01 ^bA^
WU2	1.79 ± 0.05 ^bC^	1.15 ± 0.02 ^bcB^	0.64 ± 0.02 ^eA^	10.2 ± 0.4 ^bcC^	7.5 ± 0.5 ^cdB^	5.0 ± 0.2 ^eA^	0.18 ± 0.01 ^bcC^	0.15 ± 0.01 ^abB^	0.13 ± 0.01 ^bA^
WU3	1.79 ± 0.05 ^bC^	1.04 ± 0.03 ^aB^	0.58 ± 0.01 ^dA^	10.1 ± 0.3 ^bcC^	6.7 ± 0.5 ^abB^	5.0 ± 0.3 ^eA^	0.18 ± 0.01 ^bcC^	0.16 ± 0.01 ^abB^	0.11 ± 0.01 ^aA^
WU4	1.64 ± 0.03 ^aC^	1.18 ± 0.03 ^cB^	0.56 ± 0.04 ^cdA^	10.6 ± 0.7 ^cC^	7.1 ± 0.2 ^bcB^	4.3 ± 0.2 ^dA^	0.16 ± 0.01 ^aB^	0.17 ± 0.01 ^abcC^	0.13 ± 0.01 ^bA^
WU5	1.67 ± 0.05 ^aC^	1.04 ± 0.02 ^aB^	0.43 ± 0.01 ^aA^	10.2 ± 0.5 ^bcC^	6.2 ± 0.3 ^aB^	4.1 ± 0.1 ^cdA^	0.16 ± 0.01 ^abB^	0.17 ± 0.01 ^bcdB^	0.11 ± 0.00 ^aA^

^(1)^ Means (*n* = 10) with different lowercase letters within the same column and those with different uppercase letters within the same row for the same parameter are significantly different (*p* < 0.05), according to Tukey’s HSD test.

**Table 5:** Weight gain and turbidity of cooking water for cooked noodles made from flours treated with xylanases.

Sample	Weight Gain (%)			Turbidity (ΔA h^−1^ g Flour^−1^)		
	NS	NM	NW	NS	NM	NW
C	118.1 ± 0.7 ^aA(1)^	134.1 ± 0.7 ^aB^	152.8 ± 2.1 ^aC^	0.62 ± 0.00 ^cC^	0.45 ± 0.00 ^aA^	0.48 ± 0.00 ^cB^
WE1	117.1 ± 1.2 ^aA^	140.8 ± 2.3 ^abB^	157.0 ± 0.1 ^abcC^	0.69 ± 0.00 ^fC^	0.48 ± 0.00 ^cB^	0.40 ± 0.00 ^aA^
WE2	119.0 ± 0.1 ^aA^	137.6 ± 3.8 ^abB^	164.9 ± 2.0 ^abcC^	0.66 ± 0.00 ^dC^	0.55 ± 0.00 ^gA^	0.60 ± 0.00 ^fB^
WE3	117.9 ± 1.7 ^aA^	140.3 ± 1.7 ^abB^	166.3 ± 3.1 ^bcC^	0.70 ± 0.01 ^fB^	0.56 ± 0.01 ^gA^	0.75 ± 0.01 ^iC^
WE4	117.2 ± 0.2 ^aA^	141.5 ± 1.1 ^abB^	167.8 ± 1.9 ^bcdC^	0.67 ± 0.01 ^deB^	0.48 ± 0.00 ^cA^	0.77 ± 0.00 ^jC^
WE5	124.2 ± 1.6 ^aA^	138.0 ± 0.9 ^abB^	168.4 ± 0.6 ^cdC^	0.47 ± 0.00 ^aA^	0.52 ± 0.00 ^eB^	0.76 ± 0.00 ^jC^
WU1	120.2 ± 0.3 ^aA^	143.4 ± 2.6 ^abB^	160.7 ± 0.0 ^acdC^	0.72 ± 0.00 ^gC^	0.54 ± 0.00 ^fB^	0.51 ± 0.01 ^dA^
WU2	119.1 ± 0.1 ^aA^	143.8 ± 2.3 ^abB^	156.0 ± 0.4 ^abC^	0.60 ± 0.00 ^bC^	0.45 ± 0.00 ^aA^	0.45 ± 0.00 ^bB^
WU3	117.0 ± 1.4 ^aA^	143.8 ± 5.0 ^abB^	169.2 ± 0.9 ^cdC^	0.67 ± 0.00 ^deB^	0.46 ± 0.00 ^bA^	0.72 ± 0.00 ^hC^
WU4	122.1 ± 3.9 ^aA^	139.2 ± 0.0 ^abA^	162.7 ± 8.9 ^abcB^	0.70 ± 0.01 ^fC^	0.50 ± 0.00 ^dA^	0.56 ± 0.00 ^eB^
WU5	120.4 ± 6.1 ^aA^	154.0 ± 12.4 ^bAB^	180.0 ± 1.8 ^dB^	0.67 ± 0.00 ^eC^	0.51 ± 0.00 ^deA^	0.64 ± 0.00 ^gB^

^(1)^ Means (*n* = 4) with the different lowercase letters within the same column and those with different uppercase letters within the same row for the same parameter are significantly different (*p* < 0.05), according to Tukey’s HSD test.

**Table 6:** Firmness, adhesiveness, and chewiness of cooked noodles made from flours treated with xylanases.

Sample	Firmness (N)			Adhesiveness			Chewiness (mJ)		
	NS	NM	NW	NS	NM	NW	NS	NM	NW
C	18.7 ± 0.3 ^fC(1)^	15.3 ± 0.3 ^eB^	12.2 ± 0.1 ^eA^	0.29 ± 0.03 ^cB^	0.21 ± 0.03 ^abA^	0.20 ± 0.04 ^cA^	10.2 ± 0.4 ^gC^	8.3 ± 0.4 ^fB^	6.9 ± 0.2 ^fA^
WE1	18.4 ± 0.3 ^fC^	14.9 ± 0.4 ^eB^	11.6 ± 0.3 ^deA^	0.28 ± 0.03 ^cB^	0.25 ± 0.04 ^bB^	0.18 ± 0.03 ^abcA^	9.9 ± 0.3 ^fgC^	7.7 ± 0.3 ^eB^	6.5 ± 0.3 ^efA^
WE2	17.5 ± 0.3 ^eC^	13.5 ± 0.3 ^dB^	11.3 ± 0.1 ^cdA^	0.26 ± 0.02 ^abcC^	0.19 ± 0.03 ^aB^	0.16 ± 0.02 ^abcA^	9.4 ± 0.2 ^deC^	7.4 ± 0.6 ^deB^	6.1 ± 0.2 ^deA^
WE3	16.7 ± 0.4 ^cC^	13.4 ± 0.5 ^dB^	10.8 ± 0.2 ^bcA^	0.26 ± 0.03 ^bcB^	0.17 ± 0.04 ^aA^	0.17 ± 0.01 ^abcA^	8.9 ± 0.4 ^bcC^	7.2 ± 0.4 ^cdeB^	5.8 ± 0.3 ^bcdA^
WE4	16.6 ± 0.3 ^cC^	11.9 ± 0.3 ^bcB^	10.7 ± 0.5 ^abcA^	0.26 ± 0.03 ^bcB^	0.18 ± 0.04 ^aA^	0.17 ± 0.02 ^abcA^	8.9 ± 0.2 ^bcC^	6.4 ± 0.3 ^bB^	5.5 ± 0.3 ^abcA^
WE5	15.9 ± 0.2 ^bC^	11.4 ± 0.2 ^bB^	10.6 ± 0.4 ^abA^	0.25 ± 0.02 ^abcB^	0.17 ± 0.03 ^aA^	0.15 ± 0.03 ^abA^	8.5 ± 0.3 ^abC^	6.4 ± 0.3 ^bB^	5.6 ± 0.4 ^abcA^
WU1	18.3 ± 0.2 ^fC^	13.0 ± 0.5 ^dB^	11.5 ± 0.2 ^dA^	0.29 ± 0.02 ^cB^	0.21 ± 0.03 ^abA^	0.19 ± 0.04 ^abcA^	9.6 ± 0.3 ^efC^	7.0 ± 0.4 ^cdB^	6.1 ± 0.2 ^deA^
WU2	17.3 ± 0.2 ^deB^	11.7 ± 0.2 ^bA^	11.5 ± 0.2 ^dA^	0.28 ± 0.04 ^cB^	0.19 ± 0.04 ^aA^	0.19 ± 0.05 ^bcA^	9.2 ± 0.3 ^cdeC^	6.4 ± 0.2 ^bB^	6.0 ± 0.3 ^cdA^
WU3	16.9 ± 0.3 ^cdC^	11.6 ± 0.4 ^bB^	10.6 ± 0.2 ^abA^	0.27 ± 0.03 ^bcB^	0.18 ± 0.04 ^aA^	0.18 ± 0.03 ^abcA^	9.1 ± 0.4 ^cdC^	6.5 ± 0.3 ^bB^	5.4 ± 0.3 ^abA^
WU4	15.6 ± 0.2 ^bC^	12.3 ± 0.2 ^cB^	10.4 ± 0.6 ^abA^	0.22 ± 0.03 ^abB^	0.19 ± 0.04 ^aA^	0.16 ± 0.03 ^abcA^	8.2 ± 0.1 ^aC^	6.8 ± 0.3 ^bcB^	5.2 ± 0.5 ^aA^
WU5	14.6 ± 0.5 ^aC^	9.5 ± 0.6 ^aA^	10.2 ± 0.7 ^aB^	0.21 ± 0.03 ^aB^	0.16 ± 0.02 ^aA^	0.14 ± 0.03 ^aA^	8.1 ± 0.4 ^aB^	5.3 ± 0.4 ^aA^	5.6 ± 0.4 ^abcA^

^(1)^ Means (*n* = 4) with the different lowercase letters within the same column and those with the different uppercase letters within the same row for the same parameter are significantly different (*p* < 0.05), according to Tukey’s HSD test.

**Table 7:** Resilience, cohesiveness, and springiness of cooked noodles made from flours treated with xylanases.

Sample	Resilience			Cohesiveness			Springiness		
	NS	NM	NW	NS	NM	NW	NS	NM	NW
C	0.31 ± 0.01 ^abcA(1)^	0.35 ± 0.02 ^abB^	0.38 ± 0.01 ^deC^	0.61 ± 0.02 ^bcA^	0.62 ± 0.02 ^abAB^	0.63 ± 0.01 ^bB^	0.90 ± 0.02 ^aA^	0.88 ± 0.02 ^aA^	0.90 ± 0.02 ^cA^
WE1	0.32 ± 0.02 ^bcdA^	0.33 ± 0.02 ^aA^	0.38 ± 0.02 ^cdeB^	0.60 ± 0.01 ^abA^	0.59 ± 0.01 ^aA^	0.62 ± 0.02 ^bB^	0.89 ± 0.01 ^aB^	0.87 ± 0.01 ^aA^	0.89 ± 0.02 ^bcB^
WE2	0.32 ± 0.02 ^bcdA^	0.34 ± 0.02 ^abB^	0.37 ± 0.01 ^bcdeC^	0.60 ± 0.01 ^abA^	0.61 ± 0.05 ^abA^	0.61 ± 0.02 ^abA^	0.90 ± 0.01 ^aA^	0.90 ± 0.02 ^aA^	0.88 ± 0.02 ^abcA^
WE3	0.31 ± 0.01 ^abcdA^	0.34 ± 0.01 ^abB^	0.39 ± 0.02 ^eC^	0.61 ± 0.01 ^abcA^	0.60 ± 0.01 ^aA^	0.61 ± 0.02 ^abA^	0.88 ± 0.02 ^aA^	0.89 ± 0.02 ^aA^	0.89 ± 0.02 ^abcA^
WE4	0.32 ± 0.01 ^bcdA^	0.33 ± 0.01 ^aA^	0.35 ± 0.01 ^abB^	0.61 ± 0.01 ^bcA^	0.61 ± 0.02 ^abA^	0.61 ± 0.02 ^abA^	0.89 ± 0.01 ^aA^	0.88 ± 0.01 ^aA^	0.86 ± 0.04 ^abA^
WE5	0.33 ± 0.02 ^cdA^	0.37 ± 0.01 ^cdB^	0.36 ± 0.02 ^abcdB^	0.60 ± 0.01 ^abA^	0.63 ± 0.01 ^bB^	0.61 ± 0.01 ^abA^	0.89 ± 0.01 ^aB^	0.89 ± 0.02 ^aB^	0.86 ± 0.03 ^abcA^
WU1	0.30 ± 0.01 ^aA^	0.33 ± 0.01 ^aB^	0.35 ± 0.01 ^abC^	0.59 ± 0.01 ^aA^	0.60 ± 0.02 ^aA^	0.60 ± 0.02 ^aA^	0.89 ± 0.02 ^aA^	0.90 ± 0.04 ^aA^	0.90 ± 0.01 ^cA^
WU2	0.30 ± 0.01 ^abA^	0.37 ± 0.01 ^cdC^	0.35 ± 0.02 ^aB^	0.60 ± 0.01 ^abA^	0.62 ± 0.01 ^abB^	0.59 ± 0.02 ^aA^	0.89 ± 0.02 ^aA^	0.89 ± 0.02 ^aA^	0.88 ± 0.03 ^abcA^
WU3	0.31 ± 0.01 ^abcA^	0.35 ± 0.01 ^bcB^	0.35 ± 0.01 ^aB^	0.60 ± 0.01 ^abA^	0.62 ± 0.01 ^abB^	0.59 ± 0.01 ^aA^	0.90 ± 0.02 ^aB^	0.90 ± 0.02 ^aB^	0.87 ± 0.02 ^abcA^
WU4	0.31 ± 0.00 ^abcA^	0.34 ± 0.01 ^abB^	0.36 ± 0.01 ^abC^	0.60 ± 0.01 ^abAB^	0.62 ± 0.02 ^abB^	0.59 ± 0.02 ^aA^	0.88 ± 0.02 ^aB^	0.90 ± 0.02 ^aB^	0.85 ± 0.03 ^aA^
WU5	0.33 ± 0.02 ^dA^	0.37 ± 0.01 ^dB^	0.36 ± 0.03 ^abcB^	0.62 ± 0.01 ^cAB^	0.63 ± 0.02 ^bB^	0.61 ± 0.02 ^abA^	0.89 ± 0.02 ^aA^	0.88 ± 0.03 ^aA^	0.89 ± 0.02 ^bcA^

^(1)^ Means (*n* = 4) with the different lowercase letters within the same column and those with different uppercase letters within the same row for the same parameter are significantly different (*p* < 0.05), according to Tukey’s HSD test.

## Data Availability

The original contributions presented in the study are included in the article, further inquiries can be directed to the corresponding author.

## Abbreviations

AX, arabinoxylan; WE-AX, water-extractable arabinoxylan; WE-UX, water-unextractable arabinoxylan; SRC, solvent retention capacity; SDS, sodium dodecyl sulfate.
